# Patterns of HIV or AIDS Mortality Among Older People From 1990 to 2019 in China: Age-Period-Cohort Analysis

**DOI:** 10.2196/35785

**Published:** 2022-11-17

**Authors:** Ningjun Ren, Yuansheng Li, Zhengwei Wan, Ruolan Wang, Wenxin Zhang, Emmanuel Enoch Dzakah, Junhui Zhang, Ailing Li, Song Fan

**Affiliations:** 1 School of Public Health Southwest Medical University Luzhou China; 2 Department of Health Management Center & Institute of Health Management, Sichuan Provincial People's Hospital University of Electronic Science and Technology of China Chengdu China; 3 Department of Molecular Biology and Biotechnology, School of Biological Sciences College of Agriculture and Natural Sciences University of Cape Coast Cape Coast Ghana

**Keywords:** HIV, AIDS, aging, mortality, trends, age-period-cohort model, APC

## Abstract

**Background:**

With the increasing effectiveness of antiretroviral therapy and shifting demographics, the problem of older people with HIV or AIDS is increasingly grim in China, and neglecting infection among them may cause more serious social problems, exacerbate the difficulty of controlling HIV or AIDS transmission, and increase the risk of death.

**Objective:**

We investigated the variations in the trends of Chinese mortality by age, period, and cohort, from 1990 to 2019, to reveal the relationship between age, period, cohort, and HIV burden, as well as providing guidance for resource allocation to prevent HIV-related deaths in vulnerable target populations.

**Methods:**

We extracted the HIV or AIDS mortality data from the Global Burden of Disease. The joinpoint regression model was applied to detect changes in HIV or AIDS trends. The age-period-cohort model was used to explore the age, period, and cohort effects.

**Results:**

The trends in age-standardized mortality rates in HIV or AIDS were increased in both genders, from 0.50 to 4.54/105 individuals for males, and from 0.19 to 1.43/105 individuals for females. Joinpoint regression model showed the average annual percentage change of age-standardized mortality rates was 7.0 for male and 6.4 for female individuals, showing an increasing trend. The age effect of male HIV or AIDS mortality showed a net increase of 0.59 (–0.21 to 0.38) from the ages 50-79 years. There is a gradual upward trend in the change in risk of death from HIV or AIDS for the period effect among the older population, lowest at ages 50-54 years (–0.80 for male and –0.78 for female individuals) and highest at ages 75-79 years (0.86 for male and 0.69 for female individuals). The variation of cohort effects was complex, but both genders had a nearly consistent tendency; people born in 1920-1929 had the lowest cohort effect, and those born in 1950-1954 had the highest values.

**Conclusions:**

Our study showed a marked rise in HIV mortality for both genders in China from 1990 to 2019. Aging is an important issue in current HIV prevention and control. There is an urgent need to promote HIV testing and health education. Our findings will help predict future HIV or AIDS mortality changes and identify age-specific priority populations for intervention.

## Introduction

HIV and AIDS have been prevalent in China for more than 30 years since the first case of HIV was reported in 1985 [1]. Due to the substantial number of deaths attributed to the virus, HIV or AIDS has become the most severe notifiable infectious disease accounting for the most deaths, over 18,819, in 2020 in China [2]. With the increasing effectiveness of antiretroviral therapy (ART), people with HIV are living longer [3], and HIV or AIDS has transformed from a near-uniformly fatal infection to a chronic condition [4]. As a result, some estimates indicate that nearly 50% of persons with HIV in the United States are aged 50 years and older [5]. The situation of AIDS among older people in Europe is far from satisfactory, with estimates from some European countries predicting a “silver tsunami” within the HIV community, mirroring that of the general population, with those aged 50 years or older accounting for nearly 70% of people with HIV by 2030 [6-8]. Overlooking the risk of HIV or AIDS infection among older people is a mistake. The public perception that older people were not susceptible to HIV or AIDS infection, coupled with the lack of proper sexual education [9], exacerbates HIV among the older people. This problem is increasingly grim in China.

Furthermore, previous studies mainly focused on the age distribution of morbidity or mortality, with few studies considering both time and cohort effects [10,11]. However, period effects are also crucial in influencing the onset of disease. Period effects can also be understood as the role of social and epidemiological conditions in influencing some events, including policies, medical technology, screening tools, and even disease classification criteria. Tarone et al [12] found that the rise in the incidence of breast cancer in North America in the 1980s was due to the mass use of diagnostic mammography techniques, which increased diagnostic accuracy and thus the incidence of breast cancer. Ma et al [13] and Zhang et al [11] noted that the “Four Free and One Care” policy enacted in mainland China, which expanded HIV or AIDS screening and increased attention to HIV or AIDS, led to an increase in the incidence of HIV or AIDS and a decrease in the death rate.

Moreover, the cohort effect is because people in the same birth cohort will experience the same events at the same age. Birth cohorts that experience different events at different stages of their life course have different levels of exposure to economic, behavioral, policy, and environmental risks. Nevertheless, trends in Chinese HIV or AIDS deaths by age, among older people, remain unclear, as does the relative risk due to time and cohort effects [14]. The age-period-cohort (APC) model analyzes the age, period, and cohort effect for a comprehensive analysis to clarify the answers to these questions. This study examined elderly HIV or AIDS mortality trends by age, period, and cohort. A statistical analysis of the HIV or AIDS mortality of 50-79 years old in China from 1990 to 2019 was performed. Those effects were estimated by the APC model combined with the Intrinsic Estimator (IE) algorithm [15].

Studying HIV or AIDS mortality trends in older Chinese may reveal new information about the risk factors associated with HIV or AIDS. The finding reveals the relationship between age-period-cohort, on the one hand, and HIV or AIDS burden, on the other. It also provides guidance for resource allocation to prevent HIV-related deaths in vulnerable target populations.

## Methods

China’s HIV or AIDS mortality data were extracted from the Institute for Health Metrics and Evaluation. To examine temporal trends in HIV (coded in the International Classification of Diseases, 10th Revision) mortality over the past 30 years, we used data from the Institute for Health Metrics and Evaluation, an independent global health research center at the University of Washington in the United States. Many scientists from dozens of countries around the world wrote the Global Burden of Diseases (GBD) Injuries and Risk Factor Study (GBD 2019 [16]), which used the Bayesian disease modeling meta-regression to collect data comprehensively and accurately [17]. To standardize the mortality of different observation ages, we collected the population data of each age group from the Statistical Yearbook of Population and Employment of China from 1990 to 2019. Elderly HIV or AIDS was defined according to the United Nations program on HIV or AIDS (UNAIDS) “AIDS and aging” standards [18,19].

For the requirements of the APC model, we divided the age range of 50-79 years into 6 age groups at intervals of 5 years. Individuals younger than 50 years and older than 80 years were ruled out (>80 years old already exceeds life expectancy per capita in China, and the inclusion of a population with a complex cause of death and high mortality from reduced resistance may affect the accuracy of the model). Since the purpose of our study was aimed at older patients with HIV or AIDS, after excluding Chinese patients with HIV or AIDS who are younger than 50 years and older than 79 years, the data used in our study were from the age groups of 50-54 years old to 75-79 years old. The time range of data was from 1990-2019 (with 5 years per period) for computing the age-standardized mortality rates (ASMR) and period mortality rates.

The APC models represent a classic epidemiological approach for extracting historical morbidity and mortality risk changes from cross-sectional data, termed the cohort effect [20]. As there is a linear relationship between the age, period, and cohort, it is difficult to estimate the unique setting for every age, period, and cohort effect, referred to as the unidentification problem [21,22]. Many statistical analysis algorithms were designed to solve the unidentification problem [23-26]. Fu [15,27] applied the estimable functions and the singular value decomposition of matrices to approach the estimator of the APC model, which is the most effective for the unidentification problem, named the IE.

Finally, we described the magnitude of the rates 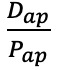 as a function of age (a), period (p), and birth cohort (c) using a log-linear model, with Poisson distribution and with the log of the person-years at risk defined as an offset of the IE method. *D_ap_* indicates the number of incidences in the “a” age group in the “p” period; *P_ap_* denotes the total number of persons in the age group “a” in period “p.”



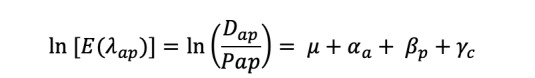



In this paper, the joinpoint regression models were performed by the Joinpoint Regression Program (version 4.3.1.0), and the age-period-cohort model analyses and graphs were conducted using APC fit in R, version 3.6.0 (R foundation for Statistical Computing). Fitting deviance, *R*^2^, and adjust *R*^2^ were used to evaluate the model; the closer the value to 1, the better the test performance.

## Results

### Mortality of HIV or AIDS in Older Chinese People

**Figure 1:**
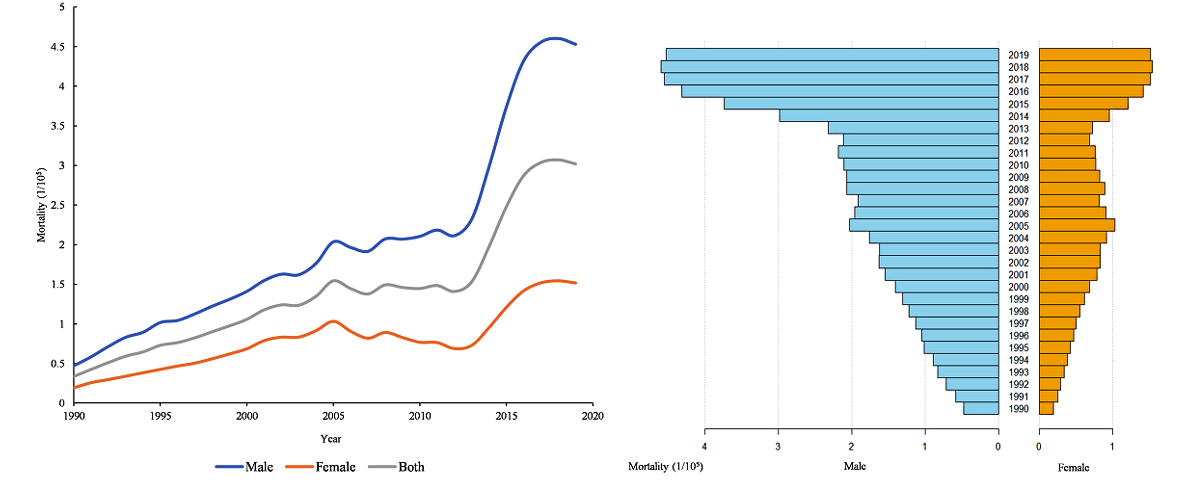
Trends in the HIV age-standardized mortality rates per 100,000 population by gender from 1990 to 2019 using the Statistical Yearbook of Population and Employment of China from 1990 to 2019 for the age-standardized population.

In Figure 1, ASMR for HIV or AIDS by gender from 1990 to 2019 was shown. HIV or AIDS ASMRs showed increasing trends from 0.50 to 4.51/10^5^ individuals for male and 0.19/10^5^ to 1.45/10^5^ for female populations, slightly decreasing after 2018. ASMR increased 8.91-fold for male and 7.31-fold for female populations over the past 30 years. Our results also indicated that the gap between the mortality rates for older male and female individuals was enormous, with a maximum of 3.36 times that of female individuals in 2013.

### HIV or AIDS Mortality Trend Variation in the Age, Period, and Cohort

**Figure 2:**
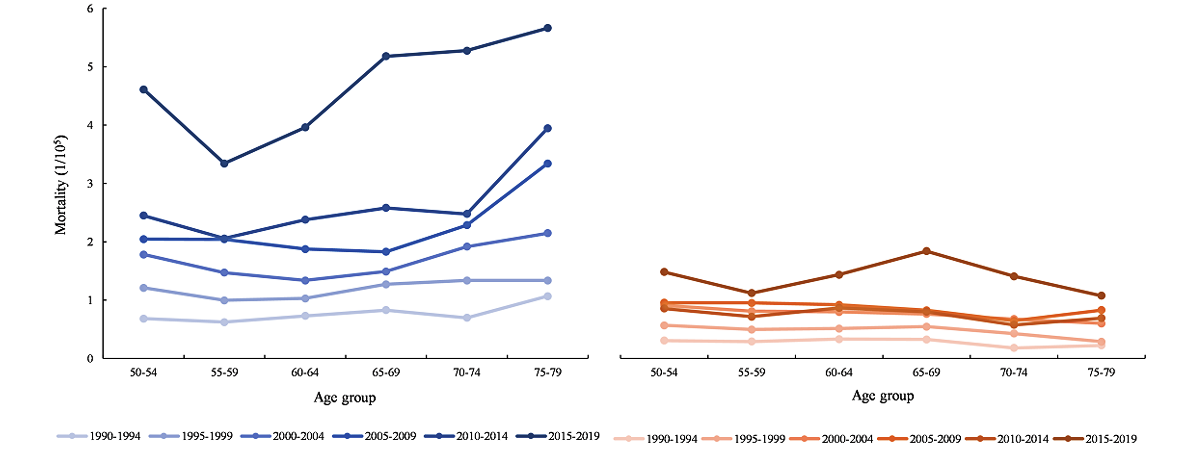
HIV mortality rates of different age groups (50-54 years old to 75-79 years old) in each period (1990-1994, 1995-1999, 2000-2004, 2005-2009, 2010-2014, and 2015-2019) are shown (male populations represented by blue lines and female populations by red).

The HIV or AIDS mortality trend variation among 50-79 years age groups of different genders in China between 1990 and 2019 is shown in Figure 2. Regardless of the period, almost all groups had insignificant changes, especially females. Only males between 2010-2014 and 2015-2019 groups showed increased HIV or AIDS mortality with age.

**Figure 3:**
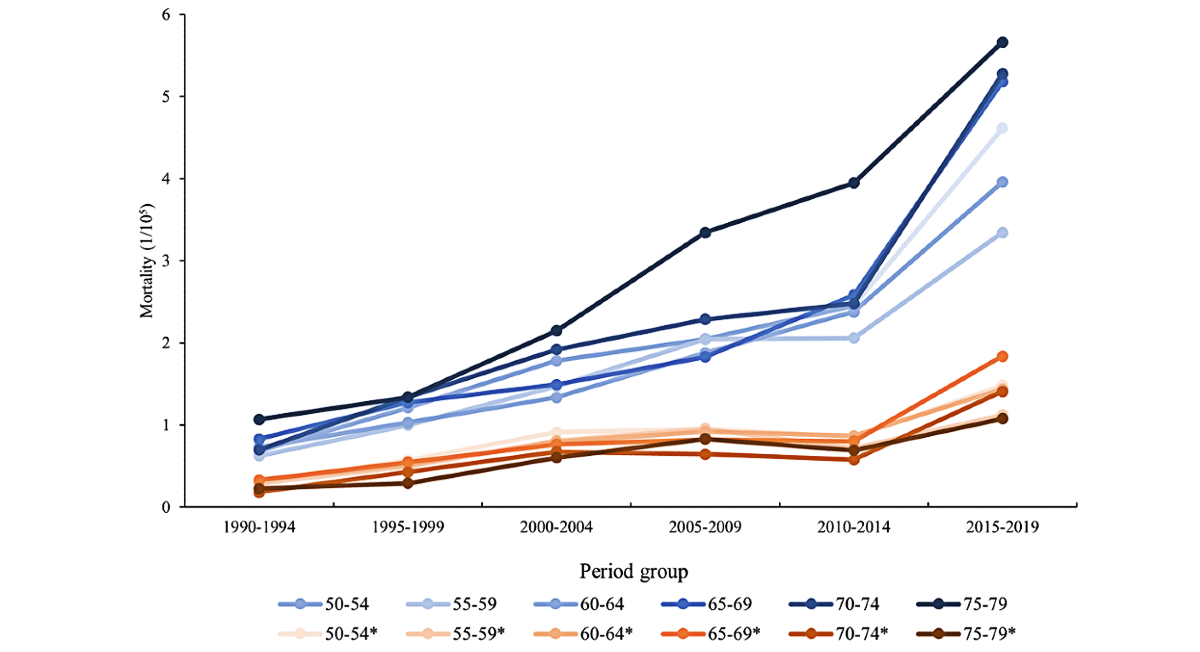
Age-adjusted mortality rates of HIV-infected persons per 100,000 person-years among men and women by age groups, 1990-2019 (adjusted to the data of the 6th population census of China in 2010 as the standard population).

The variations in the HIV or AIDS mortality rates of different age groups during the decades from 1990-2019 are shown in Figure 3. There was a significant increase in HIV or AIDS mortality regardless of age or gender. The 75-79 years age groups showed the highest mortality rate (5.66/10^5^) in males, and all groups of older males with AIDS had higher mortality rates than female groups. The male ASMR (5.66/10^5^) was over 5 times more than that of the female groups（1.08/10^5^）at ages 75-79 years from 2015-2019.

**Figure 4:**
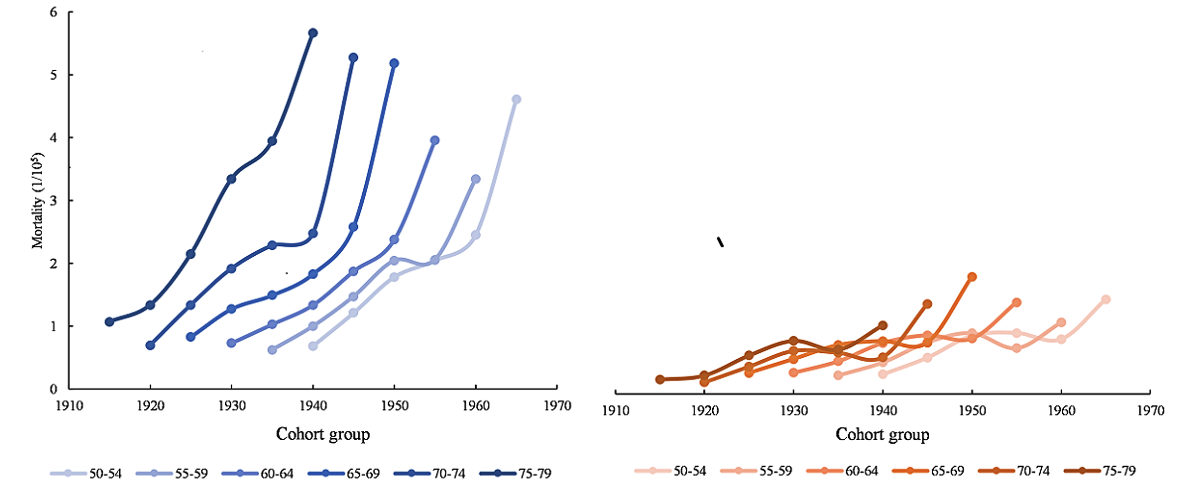
Age-specific HIV mortality in different Chinese cohorts aged over 50 years (per 100,000 people): HIV mortality rates of different age groups (50-54 to 75-79 years old) in each cohort (1915-1919, 1920-1924, 1925-1929, 1930-1934, 1935-1939, 1940-1944, 1945-1949, 1950-1954, 1955-1959, 1960-1964, and 1965-1969).

The effect of birth cohort on ASMR of HIV or AIDS among Chinese of different age groups is shown in Figure 4. The earlier the birth cohort, the higher the HIV or AIDS mortality rate. Across all cohorts, HIV mortality fluctuated more with the birth cohort, especially for males.

### Trends in the Joinpoint Regression Analysis Result

Table 1 shows the joinpoint regression analysis results of the changing trend of the death rate of patients with HIV or AIDS in China, whose age was older than 50 years in different genders, by age group during the observation period. The trends, size, and statistical significance of the mortality of HIV or AIDS in different age groups during different observation periods are described.

Over the monitoring period, trends in mortality in different age groups can be broadly divided by gender into 2 categories. Older male HIV or AIDS mortality increased over time in all age groups, with slight differences in the rate of increase between periods and mortality rates stabilizing after 2016 in most groups. All age groups saw the most significant increase from 2012 to 2016, with the 70-74 years age group exhibiting the highest APC of 30.3%, whereas the trend for older female individuals increased, then decreased, and increased again.

The first period of growth was roughly 1990-2004 with an APC of around 10%, whereas the 70-79 years age group grew by more than 15%. From 2004 to 2013, the HIV or AIDS mortality rate for older female groups decreased with an APC of around –4%. The second segment grew more significantly than the first. Similar to male groups, all APC was greater than 18% over the period 2013-2016, particularly among the 65-74 years age group, where the average annual percentage change was significant, more than 35%.

**Table 1:** The trend in HIV mortality age-standardized mortality rates for the age>50 years in all genders during 1990-2019.

Cohort and age range			Joinpoint regression analysis																										
			Trend 1				Trend 2					Trend 3					Trend 4					Trend 5					AAPC^a^		
			year		APC^b^		year		APC		year			APC		year			APC		year			APC		(%)			95% CI
**Male**																													
	adjusted	1990~2005		7.8^c^		2005~2013		2.2^c^		2013~2016			23.4^c^		2016~2019			0.4		—^d^				—	7.0^c^			(5.9~8.0)	
	50-54 years	1990~2002		10.7^c^		2002~2012		1.1		2012~2016			20.9^c^		2016~2019			2.4		—				—	7.8^c^			（6.6~8.9）	
	55-59 years	1990~2006		8.8^c^		2006~2013		–0.6		2013~2016			17.8^c^		2016~2019			4.3^c^		—				—	6.9^c^			（5.7~8.0）	
	60-64 years	1990~2013		5.7^c^		2013~2016		20.1^c^		2016~2019			–4.0		—				—	—				—	6.1^c^			（4.9~7.2）	
	65-69 years	1990~2012		4.5^c^		2012~2016		23.6^c^		2016~2019			0.1		—				—	—				—	6.5^c^			（5.4~7.7）	
	70-74 years	1990~2001		12.2^c^		2001~2013		1.6^c^		2013~2016			30.3^c^		2016~2019			2.0		—				—	8.3^c^			（6.9~9.8）	
	75-79 years	1990~2010		8.3^c^		2010~2013		–3.3		2013~2016			18.1^c^		2016~2019			–3.3		—				—	6.7^c^			（5.1~8.4）	
**Female**																													
	adjusted	1990~2004		10.4^c^		2004~2013		–3.4^c^		2013~2016			25.9^c^		2016~2019			1.5		—				—	6.4^c^			(5.2~7.6)	
	50-54 years	1990~2004		10.8^c^		2004~2012		–4.9^c^		2012~2016			18.9^c^		2016~2019			2.3		—				—	6.4^c^			（5.2~7.5）	
	55-59 years	1990~2005		10.4^c^		2005~2013		–6.0^c^		2013~2016			19.4^c^		2016~2019			2.9		—				—	5.7^c^			（4.0~7.4）	
	60-64 years	1990~2005		9.0^c^		2005~2012		–4.2^c^		2012~2016			19.5^c^		2016~2019			–2.9		—				—	5.8^c^			（5.0~6.6）	
	65-69 years	1990~2002		9.8^c^		2002~2013		–1.0		2013~2016			35.7^c^		2016~2019			2.2		—				—	7.1^c^			（5.5~8.7）	
	70-74 years	1990~2001		16.9^c^		2001~2013		–2.8^c^		2013~2016			36.1^c^		2016~2019			7.4^c^		—				—	9.0^c^			（7.0~11.2）	
	75-79 years	1990~1996		1.6		1996~2005		15.6^c^		2005~2013			–4.1^c^		2013~2016			18.6^c^		2016~2019			2.7		5.9^c^			（3.7~8.1）	

^a^AAPC: average annual percentage change.

^b^APC: annual percentage change.

^c^Indicates that the APC and AAPC are significantly different from zero at the alpha=.05 level.

^d^Not applicable.

### APC Model Analysis Results of HIV or AIDS Mortality

**Figure 5:**
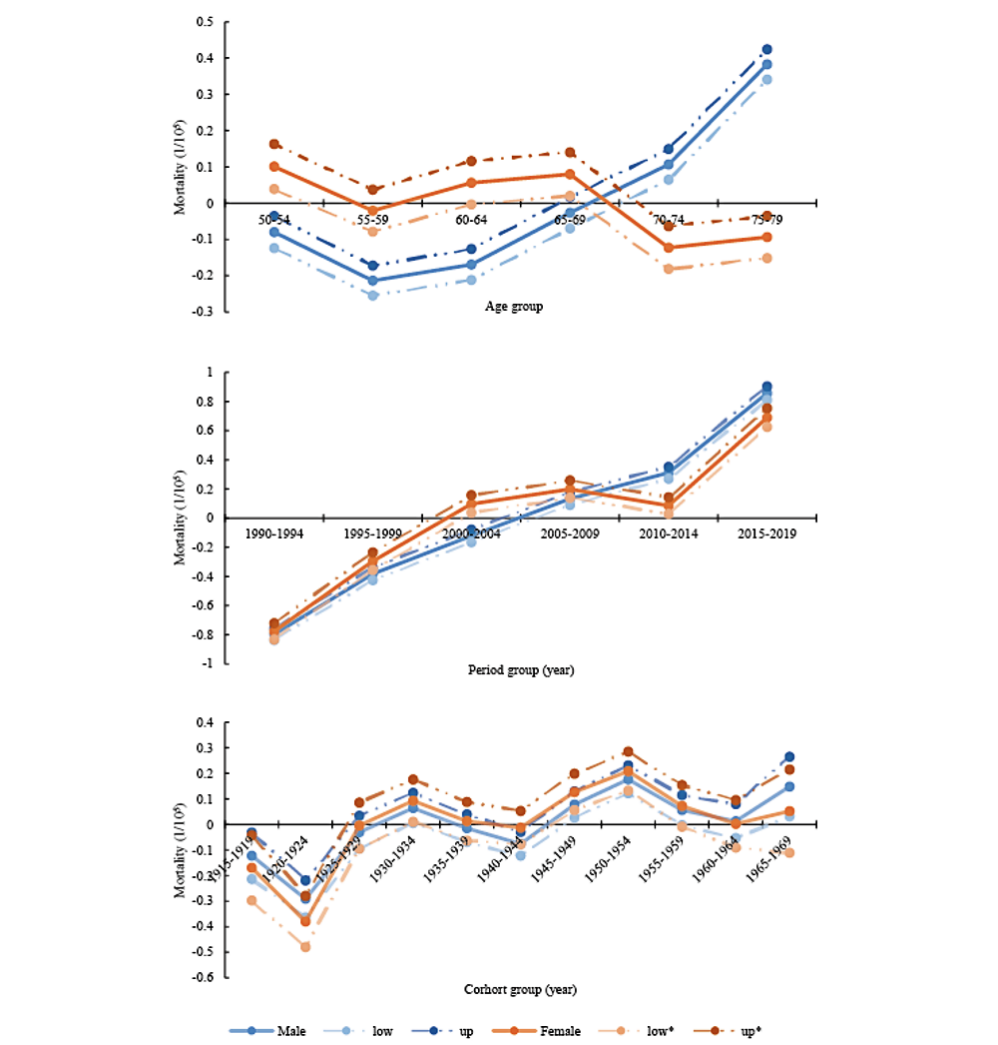
The age-period-cohort effect and 95% CI.

In this study, by fitting the age-period-cohort model, the IE algorithm was used for quantitative analysis of China’s 1990-2019 elderly HIV or AIDS deaths among different age groups and periods. The result of the analysis of HIV or AIDS mortality is shown in Table 2 and Figure 5.

The age effect of male HIV or AIDS mortality showed a net increase of 0.59 (–0.21 to 0.38), from the age of 50-79 years; using the lowest value (55-59 years age group) of the male age effect as a reference, the highest value (75-79 years age group) is 1.81 times higher. The female population’s effect was more complex than that of males, with the maximum occurring in the 65-69 years age group, and the minimum in the 70-74 years age group with less fluctuation.

According to period effects, there is a significant upward trend in the risk of death from HIV or AIDS among older people of both genders. Female groups had a slight decline after 2005 and then an increase. The risk of death is lowest at ages 50-54 years (male: –0.80; female: –0.78) and highest at ages 75-79 years (male: 0.86; female: 0.69). If the 1990 male period group is used as a reference, the period effect of HIV or AIDS mortality in 2015 increased by 5.23. This shows that the risk of HIV or AIDS deaths among older Chinese males increased by 522.61% over 30 years. Meanwhile, using the 1990 female population as the reference group, the period risk of HIV or AIDS deaths among older Chinese female individuals increased by 441.83% over 30 years.

According to the analysis of cohort effects, the mortality rates of male and female individuals living with HIV or AIDS have almost identical trends with complex and fluctuating variations similar to waves. The 1920-1924 period had the lowest cohort effect (male: –0.29; female: –0.38) on mortality risk from HIV or AIDS. Using the male population’s lowest cohort effect (1920-1924) as a reference, the highest cohort effect risk (1950-1954) of death was 1.35. The female cohort effect has 2 peaks, occurring in cohorts 1950-1954 and 1965-1969. However, the cohort effects were not statistically different (*P*>.05; Table 2).

**Table 2:** The age-period-cohort model analysis results of HIV mortality.

Cohort and age range		Coefficient	SE	*P* value	Period	Coefficient	SE	*P* value	Cohort	Coefficient	SE	*P* value
**Male^a^**												
	50-54^b^ years	–0.0798	0.0226	.003	1990-1994^b^	–0.7965	0.0203	<.001	1915-1919^b^	–0.1228	0.0466	.02
	55-59^b^ years	–0.2136	0.0211	<.001	1995-1999^b^	–0.3831	0.0218	<.001	1920-1924^b^	–0.2915	0.0366	<.001
	60-64^b^ years	–0.1692	0.0218	<.001	2000-2004^b^	–0.1217	0.022	<.001	1925-1929	–0.0307	0.0328	.36
	65-69 years	–0.0264	0.0219	.25	2005-2009^b^	0.1332	0.0217	<.001	1930-1934^b^	0.0644	0.0302	.049
	70-74^b^ years	0.1067	0.0215	<.001	2010-2014^b^	0.3109	0.0208	<.001	1935-1939	–0.0146	0.0275	.60
	75-79^b^ years	0.3824	0.0213	<.001	2015-2019^b^	0.8572	0.0235	<.001	1940-1944^b^	–0.076	0.0238	.006
	—^c^	—	—	—	—	—	—	—	1945-1949^b^	0.0779	0.0261	.009
	—	—	—	—	—	—	—	—	1950-1954^b^	0.1764	0.0278	<.001
	—	—	—	—	—	—	—	—	1955-1959	0.056	0.0299	.08
	—	—	—	—	—	—	—	—	1960-1964	0.013	0.0338	.71
	—	—	—	—	—	—	—	—	1965-1969^b^	0.148	0.0595	.02
**Female^d^**												
	50-54^b^ years	0.1007	0.0316	.006	1990-1994^b^	–0.776	0.0284	<.001	1915-1919^b^	–0.17	0.0651	.02
	55-59 years	–0.0206	0.0295	.496	1995-1999^b^	–0.2952	0.0304	<.001	1920-1924^b^	–0.3801	0.0512	<.001
	60-64 years	0.0561	0.0304	.08	2000-2004^b^	0.0971	0.0308	.006	1925-1929	–0.0048	0.0458	.92
	65-69^b^ years	0.0799	0.0306	.019	2005-2009^b^	0.1991	0.0303	<.001	1930-1934^b^	0.0929	0.0422	.04
	70-74^b^ years	–0.1227	0.0300	<.001	2010-2014^b^	0.0857	0.0291	.01	1935-1939	0.0125	0.0384	.75
	75-79^b^ years	–0.0934	0.0297	.006	2015-2019^b^	0.6893	0.0328	<.001	1940-1944	–0.0124	0.0333	.72
	—^c^	—	—	—	—	—	—	—	1945-1949^b^	0.1271	0.0365	.003
	—	—	—	—	—	—	—	—	1950-1954^b^	0.2084	0.0389	<.001
	—	—	—	—	—	—	—	—	1955-1959	0.0722	0.0417	.10
	—	—	—	—	—	—	—	—	1960-1964	0.0022	0.0473	.96
	—	—	—	—	—	—	—	—	1965-1969	0.052	0.0832	.54

^a^*R*^2^=0.9981; adjusted *R*^2^=0.9957.

^b^Indicates that age, period, and cohort effects are significantly different from zero at the alpha=.05 level.

^c^Not applicable.

^d^*R*^2^=0.9941; adjusted *R*^2^=0.9867.

## Discussion

### Principal Findings

Due to the increased effectiveness of ART, life expectancy has increased for people with HIV. Although disparities in life expectancy among people with HIV continue to persist, there is an increasing prevalence of people with HIV at 50 years of age and older [28]. However, this study showed that elderly HIV or AIDS mortality rates in China increased from 1990 to 2019, with ASMR ranging from 0.50/10^5^ to 4.54/10^5^ for male and 0.19/10^5^ to 1.43/10^5^ for female individuals. In addition to the aging population, a proportion of HIV infections occurs in older persons [4,29,30], exacerbating the severity of the HIV epidemic in the older people. Our results indicated that elderly HIV mortality in China increased rapidly, especially in male individuals (average annual percentage change=7.0). Furthermore, the ASMR showed that the mortality rate was more pronounced for male individuals as they get older, especially at 75-79 years old, but for female individuals, it peaked at 65-69 years old. It may be because with the increasing efficiency of antiretroviral therapy, the age of survival of patients who have AIDS can reach 77.3 years, which is the average life expectancy of the Chinese population [31] regardless of whether they die of diseases or natural causes.

The mortality rate of older male individuals was 2-4 times that of the older female individuals [32], both in crude rates and in ASMR, which indicated a significant gender difference in the mortality of the older people with AIDS. Possible reasons for this are that the physiological functions of people older than 50 years of age have not declined, and the physical condition and sexual needs of older male individuals are still at a high level; the standard of living of mainland Chinese residents has improved in the early 21st century [9], whereas in older female individuals, incidences are mainly due to spousal transmission [33]. However, owing to the lack of sex education, these people did not have the most basic reproductive health education and had a low perception of risk, leading to the frequent occurrence of high-risk sexual behaviors [8]. Unprotected commercial sex is the main route of HIV transmission among older males [33]. Therefore, long-term, in-depth, comprehensive HIV or AIDS health education for older male individuals is essential for critical groups.

The joinpoint regression analysis showed that the mortality rates for older male individuals have continued to increase over time (at different rates per period), while for female individuals, there was a downward trend compared with male individuals from 2003 to 2013. However, HIV or AIDS mortality rates also increased more slowly during this period compared with other periods. In 2004, the Chinese government announced its “Four Frees and One Care” policy [1], which may reduce HIV- or AIDS-related mortality or a reduction in the rate of increase. The policy has increased ART facilities from 671 in 2004 to 3733 in 2013, facilitating access to standardized ART for the HIV or AIDS population. It also strengthens the cooperation between medical institutions and the Centers for Disease Control and Prevention, continuously adjusts the types of antiviral drugs and treatment standards according to the actual ART needs of each region, and operates and establishes a system for the procurement, supply, and funding of relevant drugs.

To present more realistic results, the APC model was used to divide the influencing factors into age, period, and cohort. Age is one of the most important demographic factors affecting HIV mortality, and many surveys have shown that ages older than 40 years are strongly associated with mortality from AIDS-related diseases [34,35]. The age effect in the change of HIV or AIDS mortality among Chinese older male individuals reflects a quantitative relationship that the higher the age, the larger the effect coefficient, with the most significant age effect coefficient of 0.38 for the 75-79 years group, indicating that the high-risk group for death among Chinese older men with HIV is still people in the higher age group. In contrast to male individuals, the risk of death among older female individuals with HIV is generally decreasing. However, there is a slight increase between 55 and 69 years. Therefore, prevention and control for female populations should focus on the 50-69 years age group.

According to the analysis of the period effect on HIV mortality, there was a net increase of 1.653 from 1990-1994 to 2015-2019. Such rapid growth may suggest that the period effect is an essential factor influencing HIV- or AIDS-related deaths in older people. The continuous improvement of the quality of life and the neglect of the sexual needs of older people by their families will lead to unsafe sexual behaviors [6,36]. At the same time, due to the lack of sexual knowledge, older males often have the mentality of not being afraid or not caring. More unprotected commercial behaviors [37,38] increase HIV mortality risk during these periods. Hence, in this era of increasing material abundance, the trend will continue to affect older people living with HIV. Therefore, at a time of continuous economic and social progress, openness to sexuality, and significance of aging [39,40], we should use multidisciplinary approaches to curb the growing severity of HIV- or AIDS-related problems.

The cohort effect is a comprehensive indicator, and it is impacted by age and period effects. Only by fundamentally solving the above problems can we effectively reduce the mortality rate of HIV in older people. Community organizations should be focused on carrying out more sex education, especially among older male populations, enriching the cultural life of older people and promoting healthy and safe sexual attitudes [41]. More attention must be paid to HIV or AIDS education in low-income and rural areas, raising awareness about its health risks and impact on families and society in an acceptable manner [42-44]. In particular, maintaining a single sexual partner and the correct use of condoms must be the focus of education. Moreover, continuously carrying out voluntary counseling and testing, actively mobilizing the older population for HIV testing for early detection of elderly HIV, and providing timely care and effective treatment [45] are required.

### Limitations

This study also has some limitations. First, this paper only provides a descriptive analysis of the GBD 2019 database without etiological and attribution analyses. Second, we could not discuss China’s provinces and the differences between regions due to data inadequacy. Third, the results of GBD 2019 are mainly estimates obtained from calculations by combining a system dynamics model with a statistical model, which may differ from the actual observed data and cannot avoid distortion of the results. Finally, our study has ecological fallacies and unique limitations associated with the APC model (including identifiability issues and the uncertainty principle). Therefore, future large-scale cohort studies are needed to confirm the relevant hypotheses in this study.

### Conclusion

In conclusion, our study shows a marked increase in HIV mortality for both sexes in China from 1990 to 2019. These trends may be due to changes in socioeconomic growth and lifestyle in the population. The aging trend of the population is still a significant problem for HIV prevention and treatment in older people. It is essential to carry out early HIV screening and health education for people aged 50 years and older, as is urging infected individuals to receive ART as soon as possible to prevent HIV infection and reduce mortality rate. These findings may help predict future changes in HIV mortality and identify priority populations.

## Abbreviations

APC: age-period-cohort
ART: antiretroviral therapy
ASMR: age-standardized mortality rates
GBD: Global Burden of Disease
IE: Intrinsic Estimator

## Data Availability

The data set supporting the conclusions of this article are available in the GBD Data Tool repository [46].

Demographic data were collected from the Statistical Yearbook of Population and Employment of China by the National Bureau of Statistics for the calendar years 1990 to 2019 [47].
